# Comparative Effectiveness and Safety of Oral Versus Subcutaneous Semaglutide in Type 2 Diabetes Mellitus: A Systematic Review and Meta-Analysis

**DOI:** 10.7759/cureus.82497

**Published:** 2025-04-18

**Authors:** Jithin Karedath, Scott Nall, Mandeep Kaur, Adnan Lokhandwala, Yousef H Aqel, Abdelaziz Maali Abusal, Calvin R Wei, Danish Allahwala

**Affiliations:** 1 Internal Medicine, King's College Hospital National Health Service (NHS) Foundation Trust, London, GBR; 2 Medicine, Central Michigan University (CMU) School of Medicine, Saginaw, USA; 3 Hospital Medicine, Hospital Corporation of America (HCA) Capital Regional Medical Center, Tallahassee, USA; 4 Medicine, Khalifa University, Abu Dhabi, ARE; 5 Medicine, Hamad Medical Corporation, Doha, QAT; 6 Internal Medicine, Hamad General Hospital, Doha, QAT; 7 Research and Development, Shing Huei Group, Taipei, TWN; 8 Nephrology, Fatima Memorial Hospital, Karachi, PAK

**Keywords:** glucagon-like peptide-1 receptor agonist, glycemic control, meta-analysis, semaglutide, type 2 diabetes mellitus

## Abstract

Semaglutide, a glucagon-like peptide-1 (GLP-1) receptor agonist, has emerged as an important therapeutic option for type 2 diabetes mellitus (T2DM), available in both oral and subcutaneous formulations. While subcutaneous semaglutide has been available since 2017, oral semaglutide (approved in 2019) represents the first oral GLP-1 receptor agonist. This meta-analysis compared the efficacy and safety of oral versus subcutaneous semaglutide in patients with T2DM. We conducted a systematic literature search across PubMed, Embase, Cochrane Library, and Web of Science from inception to March 15, 2025. Four studies (one randomized controlled trial and three retrospective studies) with a total of 559 patients (257 receiving oral and 302 receiving subcutaneous semaglutide) met our inclusion criteria. The included studies were published from 2017 to 2024. All studies had a six-month follow-up duration. Outcomes included changes in HbA1c, body weight, and treatment discontinuation due to adverse effects. Pooled analysis indicated that subcutaneous semaglutide achieved significantly greater reductions in HbA1c compared to oral semaglutide (SMD: 0.21, 95% CI: 0.04 to 0.38) with low heterogeneity (I-square: 22%). For body weight reduction, subcutaneous semaglutide showed greater effectiveness, though the difference was not statistically significant (SMD: 0.12, 95% CI: -0.27 to 0.52) with high heterogeneity (I-square: 81%). Notably, patients receiving oral semaglutide had a significantly higher risk of treatment discontinuation due to side effects (RR: 1.79, 95% CI: 1.13 to 2.83) with no heterogeneity (I-square: 0%). While both formulations demonstrated clinical benefits, subcutaneous semaglutide appears to offer superior glycemic control with fewer treatment-limiting adverse effects. These findings likely reflect the differences in bioavailability and pharmacokinetics between the two formulations. However, the choice between formulations should consider individual patient factors, including treatment adherence, preference for injection versus oral administration, and specific comorbidities. Future studies should investigate whether modified dosing protocols could improve the tolerability of oral semaglutide while maintaining efficacy.

## Introduction and background

Type 2 diabetes mellitus (T2DM) is a chronic metabolic disorder characterized by insulin resistance and impaired insulin secretion, leading to hyperglycemia and an increased risk of microvascular and macrovascular complications [1]. The global prevalence of T2DM continues to rise, with an estimated 537 million adults affected in 2021, a figure projected to reach 783 million by 2045 [2]. Effective glycemic control is essential to reduce the risk of complications and improve long-term outcomes in patients with T2DM [3]. Among the therapeutic options available, glucagon-like peptide-1 receptor agonists (GLP-1 RAs) have emerged as an important class of medications due to their ability to improve glycemic control, promote weight loss, and confer cardiovascular benefits [4]. 

Among GLP1-RAs, semaglutide is currently the only drug available in both subcutaneous and oral formulations. Semaglutide, a GLP-1 RA, has demonstrated potent glucose-lowering effects and cardiovascular protection in patients with T2DM [5]. It acts by stimulating insulin secretion and inhibiting glucagon release in a glucose-dependent manner, slowing gastric emptying, and reducing appetite, which contributes to weight loss. Initially developed as a once-weekly subcutaneous (SC) injection, semaglutide has shown significant efficacy in improving glycemic control and reducing the risk of major adverse cardiovascular events (MACE) in large clinical trials, including the SUSTAIN program [6]. However, the need for injectable administration has posed challenges regarding patient adherence and acceptance. 

To address these barriers, an oral formulation of semaglutide was developed using the SNAC (sodium N-[8-(2-hydroxybenzoyl) amino] caprylate) absorption enhancer technology, which facilitates semaglutide absorption in the stomach [7]. The PIONEER trials have demonstrated that oral semaglutide is effective in reducing hemoglobin A1c (HbA1c) levels and body weight, with a safety profile comparable to that of the subcutaneous formulation [8]. The availability of an oral GLP-1 RA provides an attractive alternative for patients who prefer non-injectable therapies, potentially improving treatment adherence and long-term glycemic outcomes [9]. 

While both oral and subcutaneous semaglutide have been shown to improve glycemic control and reduce body weight, head-to-head comparisons are limited, and questions remain regarding their relative efficacy and safety in real-world settings. A comprehensive evaluation of the clinical outcomes associated with oral versus subcutaneous semaglutide is essential to guide treatment decisions and optimize patient care. Therefore, this meta-analysis aims to compare the efficacy and safety of oral versus subcutaneous semaglutide in patients with T2DM, focusing on glycemic control, weight reduction, and adverse events. The findings of this analysis will provide valuable insights into the comparative effectiveness of these two formulations and inform clinical practice. 

## Review

Methodology

Literature Search and Search Strategy 

A systematic literature search was conducted across PubMed, Embase, Cochrane Library, and Web of Science from inception to 15 March 2025, without language restrictions. Key search terms included ("semaglutide" OR "GLP-1 receptor agonist") AND ("oral" OR "subcutaneous") AND ("type 2 diabetes" OR "T2DM") AND ("HbA1c" OR "glycemic control" OR "weight loss"). Boolean operators (AND/OR) refined results, and Medical Subject Headings (MeSH) terms were applied where applicable. Gray literature sources, including Clinical Trials [21] and conference abstracts from the American Diabetes Association (2019-2024), were screened to identify unpublished trials. Reference lists of included studies and relevant reviews were hand-searched to minimize selection bias. The search was performed by two authors. Any disagreement between the two authors was resolved through discussion. 

Study Selection 

Studies were selected based on Population, Intervention, Comparison, Outcomes, and Study (PICOS) criteria: adults (≥18 years) with type 2 diabetes mellitus (T2DM) receiving oral semaglutide or subcutaneous semaglutide of any dose, with outcomes including changes in HbA1c, body weight, and adverse events. Eligible designs included randomized controlled trials (RCTs), observational cohorts, or post-hoc analyses with ≥12 weeks of follow-up. Exclusion criteria encompassed case reports, animal studies, non-comparative designs, and studies lacking disaggregated data for oral and subcutaneous formulations. Two independent reviewers screened titles, abstracts, and full texts, resolving discrepancies through consensus or third-party adjudication. The study selection process was documented using the PRISMA 2020 flow diagram. 

Data Extraction and Outcomes 

Data extraction followed a standardized template generated using Microsoft Excel (Redmond, USA), capturing study characteristics (author, year, design, sample size), intervention details (dose, follow-up duration), and efficacy and safety outcomes (mean change in HbA1c, weight, and adverse event rates). Efficacy outcomes were pooled using mean differences (MD) in HbA1c and weight from baseline to follow-up. Safety outcomes included adverse events leading to discontinuation of drugs. Missing data were requested from corresponding authors, and imputation methods aligned with Cochrane Handbook guidelines were applied where necessary. 

Data Analysis 

Statistical analyses were conducted using RevMan 5.4.1. Continuous outcomes were pooled via fixed- or random-effects models, reporting standard mean difference (SMD) with 95% confidence intervals (CI). Categorical outcomes were pooled using a fixed- or random-effects model, reporting risk ratio (RR) with 95% CI. Fixed- or random-effect model selection was done based on the value of I-Square. In the case of an I-square value of 50% or less, a fixed-effect model was used. Otherwise, a random-effects model was used to calculate pooled estimates. A p-value of less than 0.05 is considered statistically significant. Heterogeneity was quantified using I² statistics, with values of 30-60% indicating moderate and >60% of high heterogeneity. 

Results 

**Figure 1:**
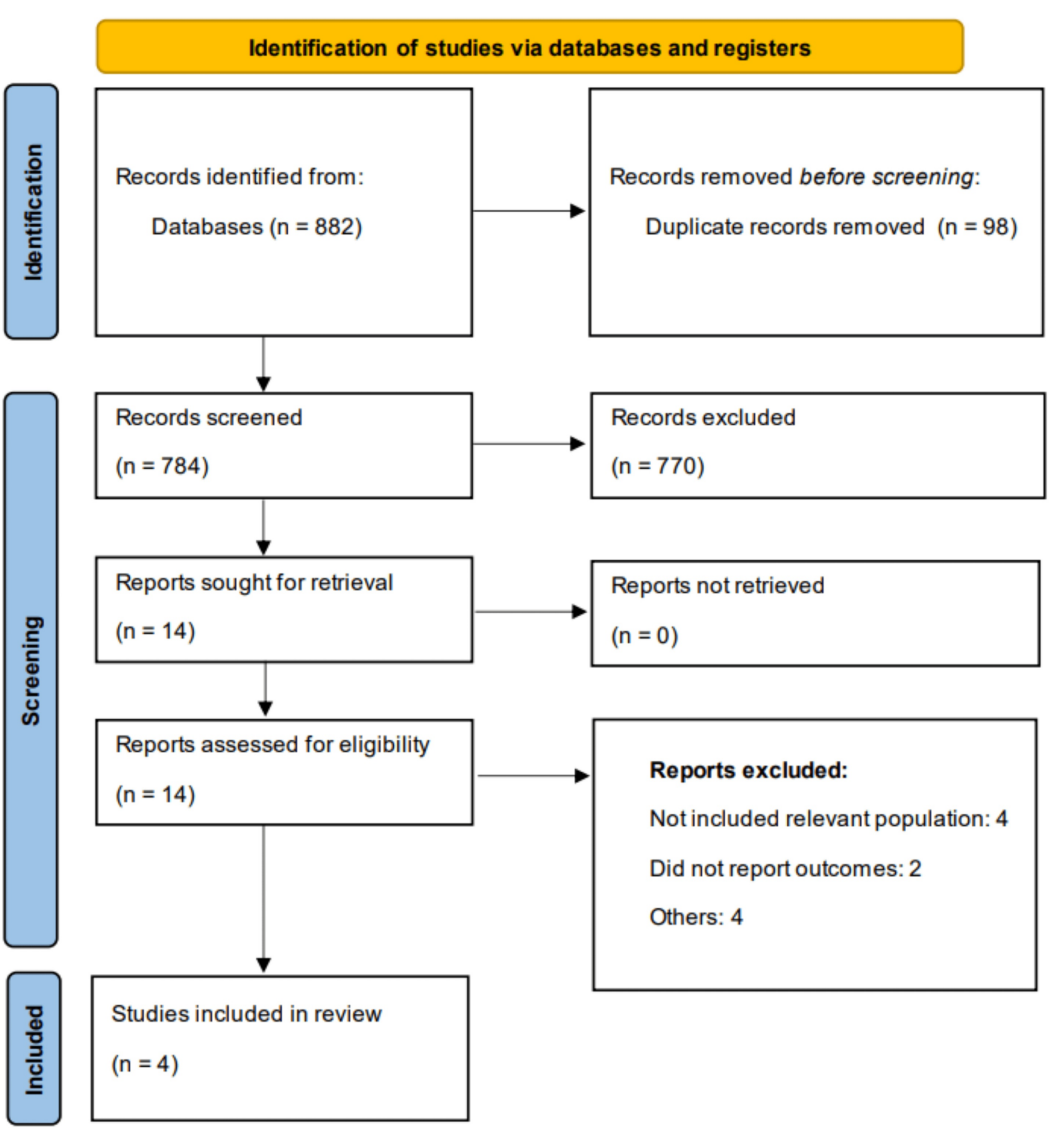
PRISMA flowchart of study selection process

Using online databases, we identified 882 studies. All references were imported to EndNote X9. After duplicates were removed, we initially screened 784 records using their title and abstracts. Overall, 14 studies were eligible for full-text screening. Finally, four papers were eligible to be part of the meta-analysis. Figure 1 shows the PRISMA flowchart of the study selection process. 

Characteristics of Included Studies 

Table 1 presents the characteristics of the included studies. Three of the four included studies were retrospective, while one RCT met the eligibility criteria. The follow-up duration of all studies was six months. The included RCT was multicenter. Included retrospective studies were conducted in Italy, Croatia, and the United Kingdom. The pooled sample size was 559 (257 in the oral group and 302 in the subcutaneous group). The majority of participants were males. The included studies were published from 2017 to 2024.

**Table 1:** Characteristics of included studies

Author ID	Year	Design	Region	Groups	Sample Size	Dose of Semaglutide	Follow-up	Males (n)	MeanAge (Years)
Chowdhury et al. [10]	2024	Retrospective	United Kingdom	Oral	53	3 to 14 mg	6 Months	26	58.45
				Subcutaneous	50	1 mg		23	56.04
Davies et al. [11]	2017	RCT	Multicenter	Oral	70	20 mg	6 Months	44	58.3
				Subcutaneous	69	1 mg		48	56.8
Formichi et al. [12]	2024	Retrospective	Italy	Oral	81	3 to 7 mg	6 Months	67	65.2
				Subcutaneous	130	0.25 to 0.5 mg		105	62.4
Klobucar et al. [13]	2024	Retrospective	Croatia	Oral	53	7 mg or 14 mg	6 Months	30	59
				Subcutaneous	53	0.5 or 1.0 mg		30	63

Meta-analysis of outcomes**

*Change in Hb1AC From Baseline* 

**Figure 2:**
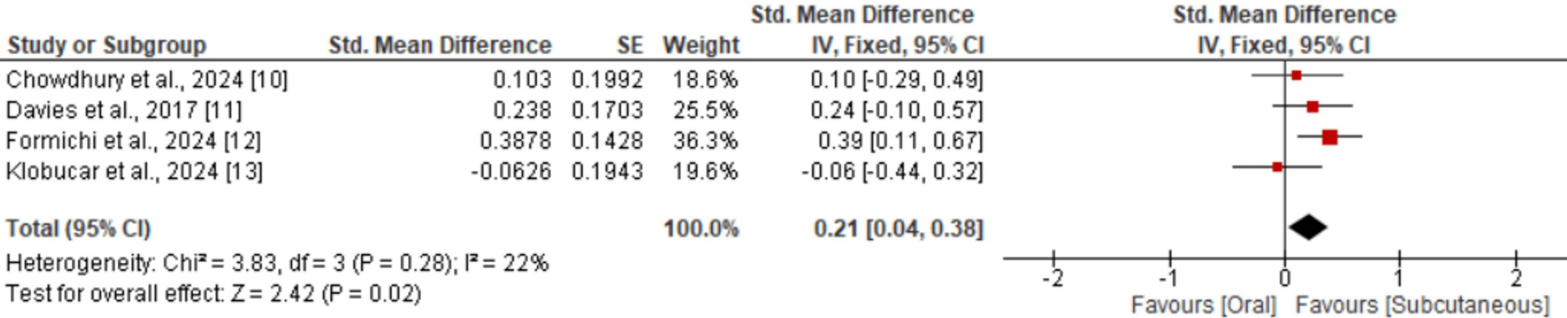
Comparison of change in Hb1Ac between oral and subcutaneous semaglutide References [10-13]

Four studies were included in the pooled analysis to compare the pooled effect of two routes of drug administration on the change in Hb1AC from baseline, and the results are shown in Figure 2. The pooled effect showed that change in Hb1AC from baseline was significantly higher in subjects receiving subcutaneous semaglutide (SMD: 0.21, 95% CI: 0.04 to 0.38). Low heterogeneity was reported among the study results (I-square: 22%).

*Change in Body Weight From Baseline* 

**Figure 3:**
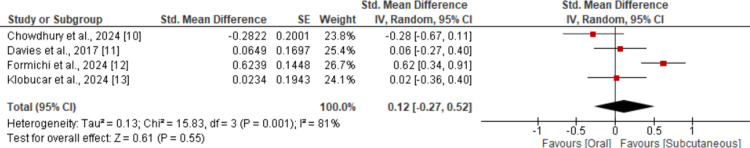
Comparison of change in body weight between oral and subcutaneous semaglutide References [10-13]

Four studies were included in the pooled analysis to compare the pooled effect of two routes of drug administration on the change in body weight from baseline, and the results are shown in Figure 3. The pooled effect showed that change in body weight from baseline was higher in subjects receiving subcutaneous semaglutide, but the difference was statistically non-significant (SMD: 0.12, 95% CI: -0.27 to 0.52). High heterogeneity was reported among the study results (I-square: 81%). 

Discontinuation of Treatment Due to Side Effects 

**Figure 4:**
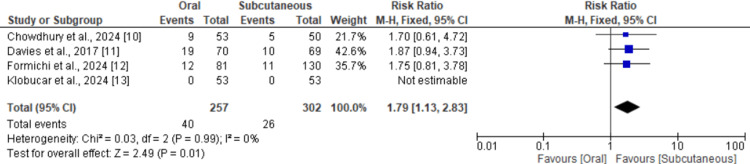
Comparison of treatment discontinuation between oral and subcutaneous References [10-13]

Four studies compared the risk of discontinuation of treatment due to side effects, and the results are shown in Figure 4. Pooled analysis indicated that the risk of discontinuation due to side effects was significantly higher in subjects receiving oral semaglutide (RR: 1.79, 95% CI: 1.13 to 2.83). No heterogeneity was reported among the study results (I-square: 0%).

Discussion 

This is the first meta-analysis that directly compared two semaglutide formulations in patients with type 2 diabetes. This study found that change in Hb1AC was significantly greater in patients receiving subcutaneous semaglutide compared to patients receiving oral semaglutide. There was no significant difference between the two groups in terms of change in body weight from baseline. 

The significantly greater reduction in HbA1c with subcutaneous semaglutide (SMD: 0.21, 95% CI: 0.04-0.38) aligns with both randomized controlled trials (RCTs) and real-world evidence. For instance, a 2024 retrospective cohort study demonstrated that subcutaneous semaglutide achieved superior HbA1c reductions compared to oral formulations (−1.5% vs. −1.1%, respectively), likely due to higher bioavailability and more stable pharmacokinetics with injectable administration [12]. Similarly, a 2021 meta-analysis of 24 trials found subcutaneous semaglutide 1 mg reduced HbA1c by −1.37% versus placebo, outperforming oral semaglutide 14 mg (−1.02%) [14]. These differences may reflect variations in dosing protocols, as oral semaglutide requires strict fasting conditions to optimize absorption [13]. Nevertheless, both formulations meet clinically meaningful thresholds for glycemic improvement, with oral semaglutide still offering advantages over traditional therapies like sulfonylureas [11]. 

While no significant inter-group difference in weight change emerged (SMD: 0.12, 95% CI: −0.27-0.52), the high heterogeneity (I²: 81%) underscores the need for patient-specific prescribing. Subgroup analyses from real-world data suggest subcutaneous semaglutide is prioritized for patients with higher baseline BMI [12], potentially amplifying weight loss effects in this population. Conversely, oral semaglutide may be equally effective in older adults, as a 2025 analysis reported superior weight outcomes in patients aged >60 years receiving oral formulations [15]. This dichotomy highlights the importance of tailoring treatment to metabolic phenotypes-a concept supported by the SUSTAIN and PIONEER trials, where subcutaneous semaglutide 1 mg reduced weight by −4.09 kg versus placebo, while oral 14 mg achieved −3.78 kg [14]. 

The higher discontinuation rate with oral semaglutide (RR: 1.79, 95% CI: 1.13-2.83) contrasts with safety profiles reported in RCTs, which found comparable gastrointestinal adverse events (e.g., nausea: 15% oral vs. 18% subcutaneous) [14]. Real-world data, however, indicate that oral formulations are often prescribed to older patients with longer diabetes duration, who may have heightened sensitivity to gastrointestinal side effects or comorbid conditions affecting tolerability. Notably, hypoglycemia risk remains low for both formulations compared to insulin or sulfonylureas [11-13], reinforcing their suitability for patients prone to hypoglycemia. Further studies should investigate whether dose escalation protocols or adjunct antiemetic therapies could improve oral semaglutide retention rates. 

The mechanism of action of oral and subcutaneous semaglutide is fundamentally similar, as both are glucagon-like peptide-1 receptor agonists (GLP-1 RAs) that act by stimulating the GLP-1 receptor to enhance glucose-dependent insulin secretion, suppress glucagon release, delay gastric emptying, and reduce appetite [16]. However, the key difference lies in their delivery and absorption. Subcutaneous semaglutide is administered via weekly injection, leading to direct absorption into the bloodstream, which ensures consistent bioavailability and prolonged therapeutic effects [17]. In contrast, oral semaglutide is formulated using SNAC (sodium N-[8-(2-hydroxybenzoyl) amino] caprylate) technology, which facilitates absorption in the stomach by increasing gastric permeability and protecting semaglutide from enzymatic degradation [18]. This results in lower and more variable bioavailability compared to the subcutaneous formulation. Due to this difference, higher doses of oral semaglutide are often required to achieve similar glycemic control as the subcutaneous form [19]. 

It might be necessary to take additional factors into account when choosing the best formulation, given the two formulations' largely comparable efficacy and safety profiles. Many patients are hesitant to start injectable pharmaceutical treatment because they are afraid of injection pain and feel like they are failing as their disease worsens. One potential drawback of taking semaglutide orally is that, in order to ensure unhindered absorption, the tablet needs to be given 30 minutes before a meal, beverage, or other medication [20]. However, it makes no difference if food or liquids were ingested before or following the injection of injectable semaglutide. Therefore, in order to best meet the needs and preferences of the patient, the ideal formulation must be chosen individually. 

The present study has certain limitations. First, only four studies have been included in this study. The predominance of retrospective studies in your meta-analysis (3 out of 4 studies) is an important limitation to acknowledge. Having only one randomized controlled trial among your included studies means that much of your data comes from study designs that are more susceptible to selection bias and confounding factors. This methodological limitation could impact the overall reliability of the findings and should be considered when interpreting the results. Second, we were not able to perform subgroup analysis, including analysis based on dose, age, gender, previous treatment, and other factors. In all included studies, the dose of oral semaglutide varies. Additionally, differences in study design and population characteristics likely contributed to variability in reported outcomes, including glycemic control, weight loss, and treatment discontinuation rates. Understanding these variations is crucial for interpreting the pooled results and assessing their applicability to real-world clinical settings. 

## Conclusions

In conclusion, this meta-analysis demonstrates that subcutaneous semaglutide achieves significantly greater HbA1c reductions compared to oral semaglutide in patients with type 2 diabetes mellitus, while effects on weight loss were comparable but with high heterogeneity. The higher treatment discontinuation rate with oral semaglutide suggests potential tolerability challenges despite its non-injectable administration advantage. These findings highlight the importance of individualized treatment selection based on patient characteristics, preferences, and treatment goals. While subcutaneous semaglutide may be preferred for those prioritizing maximal glycemic control, oral semaglutide offers a valuable alternative for injection-averse patients. Future research should focus on optimizing oral semaglutide dosing protocols to improve tolerability while maintaining efficacy and identifying patient subgroups most likely to benefit from each formulation. Both formulations represent important therapeutic options in the management of type 2 diabetes, expanding the armamentarium available to clinicians and patients.
